# Deconstructing Exceptional Responses to Immune Checkpoint Inhibition in Recurrent or Metastatic Head and Neck Carcinoma: A Site-Specific Clinical–Biological Synthesis

**DOI:** 10.3390/cancers18172790

**Published:** 2026-08-27

**Authors:** Giovanni Motta, Piero Giuseppe Meliante, Pasquale Capasso, Francesco Chiari, Giuseppe Tortoriello

**Affiliations:** 1 Otolaryngology Head and Neck Surgery Unit, Azienda Ospedaliera di Rilievo Nazionale dei Colli, Ospedale Monaldi, 80131 Naples, Italy; meliantepierog@hotmail.it (P.G.M.); pasquale.capasso@ospedalideicolli.it (P.C.); dott.giuseppetortoriello@virgilio.it (G.T.); 2 Otolaryngology, Head and Neck Unit, “Santo Spirito” Hospital, 65124 Pescara, Italy; francesco.chiari.med@gmail.com

**Keywords:** head and neck squamous cell carcinoma (HNSCC), immune checkpoint inhibitors, pembrolizumab, nivolumab, exceptional responders, stomal recurrence, tumor microenvironment, narrative review, human papillomavirus (HPV), Epstein-Barr virus (EBV)

## Abstract

**Background**: Recurrent or metastatic head and neck squamous cell carcinoma (R/M HNSCC) is an aggressive malignancy with a historically poor prognosis. Although immune checkpoint inhibitors (ICIs) targeting the PD-1/PD-L1 axis have established a new first-line standard of care, durable clinical benefit is restricted to a minority of patients, while primary or acquired resistance remains a major clinical challenge. This narrative review aims to provide a conceptual clinical–biological synthesis of immunotherapy in R/M HNSCC through an anatomical and biomarker-driven lens, with a focus on characterizing potential shared features of “exceptional responders”. **Methods**: A targeted narrative literature search was conducted across PubMed/MEDLINE and to identify key clinical trials and representative clinical reports of exceptional response to ICIs in R/M HNSCC. The literature was not systematically synthesized to construct a conceptual clinical–biological framework of response and resistance. **Results**: Landmark clinical data confirm durable survival benefits with frontline pembrolizumab-based regimens in selected PDL-1 positive populations compared to historical chemotherapy. Qualitative appraisal of illustrative cases and translational cohorts suggests a potential biological hypothesis: exceptional responders, defined as patients achieving unexpected, multi-year complete radiological, pathological, or metabolic remissions, often align with a favorable confluence of a pre-existing “hot” or inflamed tumor microenvironment, preserved antigen presentation machinery, and elevated antigenic novelty driven by viral oncoproteins (HPV, EBV) or high mutational/indel burdens. Conversely, primary and acquired resistance are conceptually associated with defects in antigen processing, immunosuppressive cellular barriers, and alternative immune checkpoints. **Conclusions**: Immunotherapy has fundamentally transformed R/M HNSCC management, yet exceptional single-agent responses remain rare. Rather than a definitive or proven biological biomarker, the proposed blueprint represents an integrative hypothesis highlighting the complex interplay of baseline immune inflammation, genomic features, and viral drivers. Translating these observations into broader clinical benefit will require validated biomarker-driven personalization and rationally designed combination regimens.

## Simple Summary

Recurrent or metastatic head and neck squamous cell carcinoma (R/M HNSCC) remains an aggressive disease with historically poor survival rates. Although the introduction of immune checkpoint inhibitors (ICIs) targeting the PD-1/PD-L1 pathway has revolutionized the treatment landscape, only a minority of patients achieve long-term survival. This review explores the clinical and biological profiles of exceptional responders to identify the key factors driving therapeutic success. We analyze how viral drivers (such as HPV and EBV), tumor microenvironment dynamics across anatomical sites, and genomic markers (such as frameshift insertions and deletions) shape immune responsiveness. Additionally, we address the mechanisms of immunotherapy resistance and outline future clinical strategies, such as next-generation checkpoint inhibitors, antibody-drug conjugates, and cancer vaccines, advocating for a transition toward personalized, biomarker-driven oncology.

## 1. Introduction

Malignancies of the head and neck represent a significant global health burden, comprising a heterogeneous group of cancers that arise from the mucosal surfaces of the oral cavity, pharynx, larynx, and sinonasal tract [1]. Structurally and genetically diverse, head and neck squamous cell carcinoma (HNSCC) is characterized by a complex mutational landscape predominantly driven by chronic carcinogen exposure (tobacco and alcohol) or high-risk oncogenic viral infections [2].

Historically, the treatment paradigm for patients presenting with recurrent or metastatic (R/M) HNSCC ineligible for curative local therapies (surgery or re-irradiation) was anchored to aggressive systemic cytotoxic chemotherapy. The EXTREME regimen—combining a platinum doublet (cisplatin or carboplatin) with 5-fluorouracil (5-FU) and the anti-epidermal growth factor receptor (EGFR) IgG1 monoclonal antibody cetuximab—served as the undisputed international benchmark for over a decade [3]. While this protocol represented a major milestone at its inception, it offered a median overall survival (OS) of merely 10.1 months and was plagued by profound toxicities, frequently necessitating dose reductions or treatment discontinuation [3].

The profound biological realization that HNSCC is an intensely immunosuppressive disease opened the door to modern immuno-oncology [4]. The tumor microenvironment (TME) of HNSCC is uniquely hostile, defined by high tumor heterogeneity, severe structural down-regulation of human leukocyte antigen (HLA) class I molecules, and an oppressive array of soluble and cellular suppressive factors [5]. The therapeutic landscape has thus shifted from non-specific cytotoxic destruction to targeted immune reactivation. However, while immune checkpoint inhibitors (ICIs) targeting the PD-1/PD-L1 axis have reshaped standard-of-care options, their clinical utility remains constrained by variable response rates and distinct immune-related adverse events.

Despite clear regulatory milestones, a critical clinical dichotomy persists: while a minority of patients achieve exceptional, highly durable multi-year remissions, approximately 60% to 70% of individuals exhibit primary resistance to immune checkpoint inhibitors (ICIs), and a significant proportion of initial responders eventually develop acquired resistance [6]. To establish a clear clinical framework for this narrative review, we explicitly define “exceptional responders” as those rare patients who achieve complete radiographic, pathological, or metabolic remissions, or who experience an unexpected, highly durable survival exceeding twice the median overall survival (OS) of the corresponding index clinical trial population. Gaining a granular understanding of the clinical and biological traits that distinguish “exceptional responders” from non-responders remains a paramount challenge in clinical oncology.

This comprehensive review synthesizes the current state of the art of systemic immunotherapy in head and neck oncology, analyzes an expanded cohort of exceptional real-world clinical cases to decode the mechanisms behind sustained complete remissions, critically evaluates the role of oncogenic viruses (human papillomavirus, HPV; and Epstein-Barr virus, EBV) and anatomical subsites in modulating immune geography, analyzes the complex biomarkers driving treatment failure, and evaluates emerging next-generation combination strategies to optimize personalized patient care.

## 2. Materials and Methods

### 2.1. Search Strategy and Information Sources

To ensure a comprehensive and balanced perspective for this state-of-the-art narrative review, a thorough literature search was executed across three primary biomedical databases: PubMed/MEDLINE, Embase, and the Cochrane Central Register of Controlled Trials (CENTRAL). The search window spanned from 1 January 2008, (marking the publication of the historical EXTREME paradigm) up to June 2026, ensuring the integration of long-term survival updates and the latest clinical case reporting.

The literature retrieval strategy was structured using a broad combination of controlled vocabulary and free-text terms to cover all relevant clinical domains. We targeted the specific anatomical regions by combining terms such as Head and Neck Neoplasms, Squamous Cell Carcinoma of Head and Neck, head and neck cancer, oropharyngeal carcinoma, laryngeal cancer, nasopharyngeal carcinoma, stomal recurrence, and peristomal. These terms were intersected with specific therapeutic agents and drug classes, including Immunotherapy, Immune Checkpoint Inhibitors, pembrolizumab, nivolumab, toripalimab, anti-PD-1, and anti-PD-L1. Finally, to isolate the desired clinical and biological outcomes, the search incorporated descriptors such as Exceptional Responders, complete response, long-term survival, case report, tumor microenvironment, and biomarkers.

These thematic clusters were combined using standard Boolean operators (AND/OR) to narrative query strings used to identify relevant literature across database fields. Given the qualitative narrative design of this work, formal systematic screening protocols and PRISMA flow diagrams were not implemented. To minimize potential omissions, manual secondary screening was conducted on the reference lists of all retrieved landmark phase III clinical trials, systematic reviews, and international clinical practice guidelines published by organizations such as the National Comprehensive Cancer Network (NCCN) [7] and the European Society for Medical Oncology (ESMO).

### 2.2. Eligibility Criteria

Articles were selected based on qualitative narrative criteria to maintain high translationally relevant value:

- Inclusion Criteria:

1. Randomized phase II or III clinical trials evaluating anti-PD-1/PD-L1 monotherapy or combination regimens in patients with R/M HNSCC.
2. Peer-reviewed, well-documented clinical case reports, case series, or institutional cohorts focusing on “exceptional responders” (defined conceptually as patients achieving complete radiographic/pathological response or unexpected multi-year survival exceeding twice the median OS of the index trial population).
3. Translational or basic science studies evaluating human HNSCC tumor microenvironments, specific spatial cellular architectures, genetic predictive biomarkers (CPS, TMB, GEP, indels), and validated mechanisms of immune evasion.
4. Studies published in the English language in peer-reviewed scientific journals.

- Exclusion Criteria:

1. Non-peer-reviewed articles, conference abstracts without full text, editorials, and commentary pieces lacking primary data or detailed translational analysis.
2. Studies focused exclusively on non-squamous histology of the head and neck (e.g., salivary gland carcinomas, thyroid cancers, mucosal melanomas).
3. Preclinical or in vitro or animal studies that do not provide direct translational insights into human immune microenvironments or clinical checkpoint inhibitor resistance.

### 2.3. Data Extraction and Conceptual Synthesis

Given the qualitative narrative nature of this review, formal quantitative data extraction, meta-analytic pooling, and systematic risk-of-bias tools were not applied. Instead, relevant findings from clinical trials, translational studies, and illustrative case reports were qualitatively synthesized. Data were extracted to map-recurring immunological themes, specifically focusing on baseline tumor microenvironment traits, viral driver status, genomic alterations, and subsite-specific clinical behaviors, to construct a conceptual framework of response and resistance to immune checkpoint inhibition in R/M HNSCC.

## 3. State of the Art: Systemic Immunotherapy in R/M HNSCC

The clinical integration of monoclonal antibodies targeting the programmed cell death 1 (PD-1) receptor has rewritten international clinical practice guidelines for both frontline and platinum-refractory R/M HNSCC. The clinical integration of monoclonal antibodies targeting the programmed cell death (PD-1) receptor has rewritten international clinical practice guidelines for both frontline and platinum-refractory R/M HNSCC. These various ICIs exert distinct therapeutic effects across specific clinical settings: pembrolizumab serves as the cornerstone of first-line therapy (either as monotherapy in PD-L1 positive disease or combined with chemotherapy); nivolumab remains a key standard of care in the second-line platinum-refractory setting and has shown unique efficacy in localized salvage scenarios; and newer anti-PD-1 agents such as toripalimab and camrelizumab have established breakthrough efficacy specifically in EBV-driven nasopharyngeal carcinomas [4,8,9,10,11,12,13,14,15,16,17,18,19].

### 3.1. The Second-Line Breakthrough: Platinum-Refractory Disease

The initial regulatory approvals of ICIs in head and neck oncology were driven by phase III trials evaluating patients who experienced disease progression during or within 6 months of receiving platinum-based chemotherapy.

- CheckMate-141: This landmark, randomized Phase III trial evaluated the anti-PD-1 antibody nivolumab versus investigator’s choice of standard single-agent systemic therapy (methotrexate, docetaxel, or cetuximab) in 361 patients with platinum-refractory R/M HNSCC [8]. Nivolumab demonstrated a statistically significant prolongation of median OS (7.5 vs. 5.1 months; Hazard Ratio [HR] = 0.70; 95% CI, 0.51–0.96; *p* = 0.01) alongside a significantly superior safety profile, with grade 3–4 treatment-related adverse events (TRAEs) occurring in just 13.1% of the immunotherapy arm compared to 35.1% of the chemotherapy arm [8]. Long-term 3-year follow-up analyses confirmed a continuous survival tail, proving that a subset of patients derives extended, multi-year survival benefits regardless of HPV status [9].
- KEYNOTE-040: In an analogous platinum-refractory R/M HNSCC population, the randomized Phase III KEYNOTE-040 trial compared the anti-PD-1 antibody pembrolizumab against investigator’s choice standard therapy [10]. Pembrolizumab monotherapy demonstrated a significant survival advantage over chemotherapy, with a median OS of 8.4 months versus 6.9 months (HR = 0.80; 95% CI, 0.65–0.98; *p* = 0.016) [10]. Crucially, this study confirmed that clinical utility and survival benefits directly scaled with increasing PD-L1 expression, establishing the Combined Positive Score (CPS) as a vital clinical metric for optimizing patient selection in single agent regimens.

### 3.2. The First-Line Paradigm Shift

The therapeutic hierarchy of R/M HNSCC was completely reordered by the results of the KEYNOTE-048 Phase III trial [11]. This three-arm study randomized 882 untreated patients to receive pembrolizumab monotherapy, pembrolizumab combined with a platinum doublet (cisplatin or carboplatin) and 5-FU, or the standard EXTREME regimen (cetuximab plus platinum/5-FU) [11]. While PD-L1 CPS serves as a critical biomarker for single-agent pembrolizumab, it is not an absolute gatekeeper for first-line immunotherapy, as the pembrolizumab–chemotherapy combination demonstrated OS superiority over EXTREME in the unselected total population (Table 1) [11].

Long-term 5-year updates from KEYNOTE-048 confirmed that the survival rate at 48 and 60 months was roughly doubled in patients receiving pembrolizumab-containing regimens compared to those on the EXTREME protocol [12]. Specifically, the 5-year OS rate for pembrolizumab monotherapy in the CPS ≥ 20 population was 23.9% versus 9.9% for the EXTREME regimen [12].

### 3.3. Lessons from Exceptional Responders: Extended Case-Based and Molecular Synthesis

While median survival statistics from phase III trials frame regulatory milestones, individual instances of “exceptional responders” provide invaluable biological hypotheses regarding potential determinants of treatment success. By evaluating patients who achieve prolonged, multi-year complete remissions under immunotherapy for highly lethal recurrences, recent literature has offered key translational insights into candidate microenvironmental and genomic configurations associated with therapeutic clearance.

- **Reversal of High-Mortality Peristomal Stomal Recurrence.** Stomal recurrence (SR) following a total laryngectomy represents one of the most dreaded complications in head and neck surgery, characterized by rapid, diffuse infiltration of squamous cells at the junction of the amputated trachea and the cervical skin [13]. Historically, the prognosis is catastrophic, with a 3-year mortality rate approaching 100% despite combinations of radical surgery, re-irradiation, and standard salvage chemotherapy [13]. In a notable institutional study, Mesolella et al. documented a landmark case of a patient presenting with an aggressive, platinum-refractory stomal recurrence following a total laryngectomy for advanced laryngeal carcinoma [13]. While traditional therapeutic choices were entirely exhausted, the patient was initiated on a salvage protocol consisting of the anti-PD-1 human monoclonal antibody nivolumab [13]. Strikingly, the patient exhibited an exceptional clinical response, achieving a complete and durable regression of the peristomal tumor mass. This landmark outcome suggests that, in selected clinical scenarios, immune modulation may overcome adverse tissue environments inherent to heavily operated, fibrotic, and radiation-damaged dermal-tracheostomal junctions, offering a potential paradigm for remarkable recovery in stomal relapses [13].
- **Molecular Correlates of Exceptional Responses in Viral-Driven HNSCC (HPV and EBV).** Beyond isolated clinical scenarios, landmark molecular screening initiatives, such as the National Cancer Institute (NCI) Exceptional Responders Initiative, have demonstrated that long-term, multi-year complete radiological and metabolic remissions under single-agent anti-PD-1/PD-L1 therapy are frequently associated with distinct viral oncology profiles [14,20]. In human papillomavirus-positive (HPV+) oropharyngeal cancers, favorable responses to pembrolizumab or nivolumab are conceptually linked to an inherently inflamed tumor microenvironment enriched with tissue-resident memory CD8+ T cells expressing high levels of PD-1 and CD103 [21]. These viral oncoproteins generate highly immunogenic viral antigens that can foster anti-tumor immunity, provided an intact antigen-presentation machinery is maintained without downstream mutations in the *JAK1/JAK2* or beta-2-microglobulin pathways [6,14,20]. Similarly, in Epstein-Barr virus-driven (EBV+) nasopharyngeal carcinoma (NPC), exceptional responders treated with anti-PD-1 agents (such as toripalimab or camrelizumab) consistently demonstrate intense baseline PD-L1 expression, which in certain cohorts is mechanistically up-regulated by the viral onoprotein LMP1 via the NF-kappa pathway [15,16]. In these elite viral responders, complete metabolic clearance on ^18^F-FDG PET/CT is often accompanied by rapid real-time plasma clearance kinetics, where drops in EBV-DNA or HPV-ctDNA copy numbers can serve as early exploratory surrogates of deep pathological clearance [17].
- **Genomic Drivers in Carcinogen-Induced, PD-L1 Negative and Non-Oropharyngeal Malignancies.** Conversely, exceptional responses in HPV-negative, tobacco-driven subsites—such as the oral cavity, larynx, and hypopharynx—frequently align with distinct biological mechanisms. Because these tumors lack viral antigens, therapeutic clearance is often hypothesized to be influenced by high genomic instability and elevated Tumor Mutational Burden (TMB) [18]. High-throughput sequencing of exceptional responders in carcinogen-induced head and neck cancers indicates that total mutation count alone does not fully account for elite phenotypes; rather, response efficacy can be associated with a high density of tobacco-induced frameshift insertions and deletions (indels) [19]. These frameshift indels are thought to escape self-tolerance more effectively than simple missense mutations, creating entirely novel, highly immunogenic neoepitopes that stimulate robust polyfunctional T-cell responses [18,19]. Furthermore, even in patients with low or negative baseline PD-L1 expression (CPS < 1), single-agent anti-PD-1 rescue has been observed to correlate with deep clinical responses, particularly when the tumor bed exhibits an enrichment of tertiary lymphoid structures (TLS) capable of driving local B-cell and T-cell clonal expansion [18,19,22].

## 4. Site-Specific Immunotherapeutic Dynamics: Anatomical and Biological Stratification

### 4.1. Structural Mapping of Subsite Dynamics

The clinical manifestation of HNSCC is highly dependent on its specific anatomical origin, as each subsite is governed by unique baseline physiological conditions, etiochemical drivers, and architectural constraints that shape the immune topography. However, substantial interpatient biological heterogeneity exists within each anatomical subsite based on variation in smoking history, viral status, prior treatment, and individual tumor–microenvironment interactions.

In oral cavity squamous cell carcinoma (OCSCC), the microenvironment is frequently molded by chronic exposure to chemical carcinogens such as tobacco smoke and alcohol, which often align with a highly immunosuppressive, “immune-excluded”, or “cold” tumor phenotype [19]. This localized niche can be characterized by a dense infiltration of M2-polarized tumor-associated macrophages (TAMs) and CD4+FOXP3+ regulatory T cells (Tregs) that collaborate to suppress effective adaptive immunity [22]. Crucially, the physical landscape of OCSCC frequently features a rigid, cross-linked fibrotic extracellular matrix that may act as a mechanical barrier, potentially hindering therapeutic monoclonal antibodies and effector lymphocytes from successfully penetrating the tumor core, which can constrain single-agent efficacy in certain pretreated settings. Nevertheless, subsets of OCSCC displaying high baseline immune cell infiltration or distinct mutational profiles can still achieve meaningful responses [22,23].

Conversely, oropharyngeal squamous cell carcinoma (OPSCC) presents a distinct baseline paradigm when driven by high-risk human papillomavirus (HPV) infection, primarily the HPV16 strain [24]. Because these tumors often originate within the immune-competent reticular stroma of the palatine and lingual tonsils, they tend to exhibit an inflamed, lymphoid-rich, and “immune-hot” landscape [24]. The microenvironment of many HPV-positive OPSCCs is densely populated by CD103+ tissue-resident memory T cells that produce high baseline levels of interferon-gamma (*IFN-gamma*) upon reactivation [24]. This biological setting is hypothesized to favor microvascular functional integrity, allowing anti-PD-1 clones to rapidly reverse T-cell exhaustion and achieve profound, multi-focal tumor regression, contributing to the superior overall survival outcomes seen in this subgroup during major frontline trials, although it is important to note that HPV status alone does not definitively predict a superior response to checkpoint blockade [25].

Laryngeal and hypopharyngeal squamous cell carcinomas (LHSCC) represent another distinct carcinogen-driven entity that frequently shares the heavy mutational patterns of tobacco exposure, including high rates of structural *TP53* disruptions and an elevated burden of somatic frameshift insertions and deletions [26]. While this high genomic instability can generate a diverse array of neoantigens that may enhance immunogenicity, responsiveness remains contingent upon an active, uninhibited immune infiltrate. In localized disease, immunotherapy is frequently combined with radiation to achieve functional larynx-preservation goals [7]. However, when surgical salvage is unavoidable, recurrences in this area pose a severe danger. This is particularly true for stomal recurrences along the post-laryngectomy tracheostomal border, where prior operations and high-dose radiotherapy leave behind dense dermal fibrosis and a severely compromised vascular supply [13].

As extensively analyzed by several authors, the laryngeal subsite demands distinct management considerations when progressing through lines of systemic chemotherapy [7,14]. In these settings, representing a common mechanism of immune escape across various solid tumors and HNSCC subsites, the tumor cells actively exploit the PD-1/PD-L1-signaling axis to construct a dynamic shield against host immune surveillance. Crucially, their research highlights that even in heavily pretreated cohorts, targeting this axis can restore anti-tumor immunity in selected patients [7,14].

Finally, nasopharyngeal carcinoma (NPC) stands completely apart due to its strict causal association with clonal Epstein-Barr virus (EBV) infection [27]. NPC typically features a “lymphoepithelioma-like” stroma, where sheets of host immune cells wrap tightly around the malignant nests [27]. Mechanistically, the viral oncoprotein latent membrane protein 1 (LMP1) can drive this phenotype by hyper-activating host cell-signaling pathways, which frequently triggers a significant upregulation of PD-L1 across both cancer and background stromal elements [28]. This intense baseline checkpoint expression makes NPC particularly responsive to anti-PD-1 interventions, where combining ICIs with standard chemotherapy yields unprecedented progression-free survival outcomes [21,29,30,31,32].

### 4.2. Subsite Insights and Clinical Horizons

- **Oral Cavity (OCSCC):** Characterized frequently as an “immune-excluded” niche heavily enriched with CD4+FOXP3+ regulatory T cells and M2 macrophages while acknowledging substantial intertumoral and intratumoral heterogeneity [22]. It often exhibits lower baseline responsiveness in late metastatic phases due to physical stromal barriers. To break this resistance, neoadjuvant protocols are utilizing early immune checkpoint interventions to prime T cells within intact cervical lymph nodes before surgical distortion [31].
- **Oropharynx (OPSCC):** Dominated by the viral biology of HPV [24]. These lesions possess an expanded baseline population of CD103+ tissue-resident memory T cells that secrete a continuous wave of interferon-gamma, which is hypothesized to help maintain local vascular patency and enabling robust single-agent anti-PD-1 efficacy [24].
- **Larynx and Hypopharynx (LHSCC):** Highly mutagenic sites where tobacco carcinogens drive high structural indel signatures [26]. In these regions, immunotherapy is deeply integrated with radiotherapy to avoid the severe functional loss of a total laryngectomy [7]. When surgical salvage is unavoidable, the risk of localized failure remains critical. Observations from single-case clinical reports, such as Mesolella et al., suggest that aggressive peristomal stomal recurrences—traditionally considered lethal due to post-radiation fibrosis and compromised vascular supply—may, in select instances respond to nivolumab, indicating that local tissue alterations do not inevitably preclude T-cell-mediated destruction [13].
- **Nasopharynx (NPC):** Strongly associated with clonal EBV infection, where the LMP1 viral oncoprotein frequently interacts with cellular pathways to induce robust upregulation of PD-L1 on both tumors and background cells [28]. Frontline regimens combining toripalimab or camrelizumab with gemcitabine/cisplatin have established new progression-free survival records, with liquid biopsy quantitative tracking of plasma EBV DNA serving as exploratory non-invasive tool to monitor disease dynamics [29,30].

## 5. Dissecting Treatment Failure: Biomarkers and Resistance Mechanisms

### 5.1. The Complexity of Predictive Biomarkers

Predicting ICI efficacy in HNSCC requires evaluating several interconnected biological dimensions. Unlike the Tumor Proportion Score (TPS), which calculates PD-L1 expression exclusively on malignant cells, the **Combined Positive Score (CPS)** integrates the total number of PD-L1 staining cells—including tumor cells, lymphocytes, and macrophages—relative to viable tumor cells [11]. While CPS remains the primary, clinically validated diagnostic tool for frontline pembrolizumab monotherapy selection, it is not an absolute requirement for chemo-immunotherapy regimens, which show efficacy across unselected populations [11]. Furthermore, it is an imperfect biomarker that suffers from significant spatial intra-tumor heterogeneity, temporal changes induced by prior cytotoxic therapies or radiation, and inter-observer pathology variation [32].

In virus-negative HNSCC, chronic exposure to tobacco carcinogens generates a high burden of somatic single-nucleotide variants. Theoretically, a high **Tumor Mutational Burden (TMB)** correlates with increased neoantigen production and enhanced T-cell recognition [33]. However, recent data reveal that total TMB is an incomplete predictor; instead, the presence of specific frameshift insertions and deletions (indels) frequently serves as a potentially informative marker in HNSCC, as indels generate highly immunogenic, non-self novel peptides that can evade central immune tolerance [26].

Beyond structural DNA alterations, multi-gene expression panels quantified via RNA sequencing have emerged as superior predictors of clinical benefit. The 18-gene T-cell-inflamed Gene Expression Profile (GEP)—which measures transcripts related to *IFN-gamma* signaling (e.g., *STAT1*, *JAK2*, *IFNG*), T-cell homing chemokines (*CXCL9*, *CXCL10*), and cytolytic factors (*GZMB*, *PRF1*)—consistently identifies tumors that are immunologically active. This signature predicts a response to pembrolizumab independently of, and complementary to, TMB and CPS [34]. Concurrently, the cGAS-*STING* pathway serves as the primary intracellular sensor of aberrant cytosolic double-stranded DNA. In carcinogen-induced HNSCC, structural epigenetic silencing via promoter hypermethylation of the gene-encoding *STING* represents a widespread mechanism of immune evasion. This silencing prevents the initiation of spontaneous anti-tumor inflammation, rendering the tumor profoundly refractory to anti-PD-1 therapies [35].

Finally, liquid biopsies are emerging as exploratory tools for early response monitoring. Recent clinical studies demonstrate that a rapid, early clearance of plasma circulating tumor DNA (ctDNA) or viral loads (such as plasma EBV DNA copy numbers in NPC) correlates with favorable outcome trends [15]. However, complete ctDNA clearance within short initial treatment windows is not uniformly observed across all clinical responders, and liquid biopsy kinetics currently serve as exploratory surrogates rather than definitive clinical discriminators between true progression and pseudo-progression in routine practice [15].

### 5.2. Mechanisms of Primary and Acquired Resistance

- **Defects in Antigen Presentation (Antigen Blindness):** A primary mechanism of intrinsic and acquired evasion is the structural loss or transcriptional silencing of the antigen presentation machinery. This is frequently driven by somatic mutations or loss of heterozygosity (LOH) in the beta-2-microglobulin gene, or via the transcriptional downregulation of Transporter associated with Antigen Processing 1 (*TAP1*) and *TAP2* [35]. Consequently, even in the presence of high concentrations of anti-PD-1 antibodies, cytotoxic T cells frequently fail to effectively recognize tumor targets, contrasting with the molecular architecture described in exceptional responders [20].
- **The Immunosuppressive Cellular Shield:** Non-responsive (“cold” or “immune-excluded”) HNSCC tumors actively construct a cellular and metabolic barrier. This TME is heavily enriched with CD4+CD25+FOXP3+ regulatory T cells (Tregs), immature Myeloid-Derived Suppressor Cells (MDSCs), and M2-polarized tumor-associated macrophages (TAMs) [22]. These immunosuppressive cellular subsets actively secrete high concentrations of TGF-beta and IL-10, which drive the formation of a dense, collagenous extracellular matrix. This matrix physically excludes T cells from the tumor nest and induces profound, irreversible functional exhaustion in any successfully infiltrating CD8+ T cells [22,23].
- **Upregulation of Alternative Immune Checkpoints and Oncogenic Pathways:** Under the continuous selective pressure of prolonged anti-PD-1 monotherapy, HNSCC tumors frequently adapt by upregulating alternative, compensatory co-inhibitory pathways, including Lymphocyte Activation Gene 3 (LAG-3), T-cell Immunoglobulin and Mucin-Domain Containing-3 (TIM-3), and T-cell Immunoreceptor with Ig and ITIM Domains (TIGIT) [36,37]. Furthermore, tumor-intrinsic genetic alterations can actively dictate a “cold” immune phenotype. Hyper-activation of the Wnt/beta-catenin-signaling pathway leads to the transcriptional repression of chemokines like CCL4, preventing dendritic cell recruitment [37]. Similarly, *PTEN* loss or hyper-activation of the PI3K/AKT/mTOR pathway upregulates immunosuppressive cytokines, fostering an immune-excluded phenotype that is often refractory to anti-PD-1 monotherapy [37].

## 6. Synthesis: Core Commonalities of Exceptional Responders

A comparative cross-examination of the presented clinical cases suggests a working hypothesis of a strong association between exceptional response and a highly specific convergence of baseline clinical, genomic, and microenvironmental architectures.

The table below details the recurring working hypothesis of a biological blueprint shared by these elite patients (Table 2):

## 7. Current Clinical Conundrums: Navigating Pseudo-Progression and Spatial Biomarker Heterogeneity

As the therapeutic landscape of head and neck oncology shifts toward biomarker-driven precision medicine, two pivotal open questions continue to complicate routine clinical decision-making: the accurate identification of pseudo-progression and the optimal tissue-sourcing strategy for PD-L1 scoring.

### 7.1. The Diagnostic Dilemma of Pseudo-Progression vs. True Disease Progression

A significant challenge in managing recurrent or metastatic head and neck squamous cell carcinoma (R/M HNSCC) under immune checkpoint inhibition is distinguishing true disease progression from pseudo-progression [16]. Pseudo-progression represents a radiographically captured increase in tumor burden or the appearance of new lesions, which is paradoxically followed by a subsequent tumor reduction or stabilization [16,35]. Although recognized, pseudo-progression remains an uncommon event in HNSCC. Biologically, this phenomenon does not reflect therapeutic failure or tumor cell proliferation; rather, it is driven by a massive intratumoral influx and recruitment of activated Tumor-Infiltrating Lymphocytes (TILs), accompanied by localized edema, hemorrhage, and transient inflammatory remodeling within the tumor microenvironment (TME) [5,16].

In R/M HNSCC, differentiating these two entities is critical to avoid premature termination of a potentially life-saving immunotherapy [16,35]. True progression is characterized by an increase in tumor volume, which may be accompanied by site-specific clinical deterioration, though clinical worsening is not strictly required to define progressive disease. Conversely, during pseudo-progression, patients often maintain a stable or improved performance status and report overall symptomatic relief despite the radiological alert [16]. To effectively navigate this clinical dilemma, traditional RECIST 1.1 criteria [38] have been supplemented by immune-related response criteria, such as **iRECIST** [38], which was developed primarily for standardized response assessment within clinical trials rather than routine practice [38]. Under iRECIST, an initial increase in tumor size is classified as unconfirmed progressive disease (iUPD). A definitive diagnosis of confirmed progressive disease (iCPD) requires a repeat imaging assessment performed 4 to 8 weeks later, showing further progression in the target lesions or a continued increase in the burden of new lesions [38]. Furthermore, innovative diagnostic tools like circulating tumor DNA (ctDNA) kinetics and ^18^F-FDG PET/CT metabolic monitoring are emerging as vital adjuncts: a sharp decline in plasma EBV-DNA or HPV-ctDNA copies, alongside stable or decreased metabolic activity (SUVmax), may serve as promising exploratory indicators to help differentiate pseudo-progression from true progression long before structural radiological stabilization is anatomically evident [15].

### 7.2. Spatiotemporal Heterogeneity of PD-L1 Expression: Sourcing CPS from Primary vs. Recurrent Lesions

The determination of the Combined Positive Score (CPS) is the primary diagnostic tool for first-line pembrolizumab allocation in R/M HNSCC [11,32]. However, a major clinical and pathological conundrum revolves around whether the CPS should be evaluated on historical tissue retrieved from the primary surgical intervention or sourced directly from a fresh biopsy of the recurrent/metastatic lesion [32].

PD-L1 expression is inherently dynamic and highly susceptible to significant spatiotemporal heterogeneity [14]. While evaluation of the archival primary tumor is often logistically more accessible, multiple comparative analyses indicate that the concordance rate of PD-L1 expression between paired primary and recurrent head and neck specimens is suboptimal [32]. Intervening therapeutic modalities—such as platinum-based chemotherapy, cetuximab, and particularly ionizing radiation—exert profound selective pressures on the tumor architecture, frequently modulating or downregulating PD-L1 expression in the recurrent tumor beds [6,32]. Conversely, certain aggressive recurrences, such as post-laryngectomy stomal recurrences, may present an extensively remodeled, highly inflamed local microenvironment that alters the baseline CPS [13].

Therefore, whenever clinically feasible and safe, obtaining a fresh biopsy of the recurrent or metastatic lesion may offer valuable clinical insight regarding current CPS status [32]. However, performing a fresh biopsy does not invariably provide an accurate real-time immune profile, as spatial intratumoral heterogeneity, potential sampling errors, and procedural limitations can confound histological interpretation [6]. Consequently, clinicians must carefully weigh the diagnostic utility of a repeat biopsy against sampling biases and logistical constraints when tailoring treatment strategies [32].

## 8. Emerging Immunotherapeutic Strategies and Future Horizons

As the therapeutic landscape of head and neck oncology shifts, overcoming primary and acquired resistance to single-agent immune checkpoint blockage represents a major clinical imperative. Novel combinatorial paradigms, targeted delivery systems, and cell-based platforms are currently under investigation.

### 8.1. Next-Generation Checkpoint Inhibitors

Simultaneous dual or triple checkpoint inhibition represents a logical evolutionary step to circumvent compensatory resistance pathways. LAG-3 is highly co-expressed with PD-1 on exhausted TILs in HNSCC. Clinical trials are actively evaluating combinations of anti-PD-1 agents with novel anti-LAG-3 monoclonal antibodies (such as relatlimab). By concurrently blocking these two distinct, non-overlapping inhibitory mechanisms, these combinations aim to re-energize deeply exhausted T-cell clones within the mucosal TME [39]. Novel protocols are similarly exploring combination regimens utilizing anti-TIGIT (e.g., tiragolumab) or anti-TIM-3 agents to disrupt alternative checkpoint networks [40].

### 8.2. Antibody-Drug Conjugates (ADCs) and Targeted Combinations

One strategy under exploration to enhance immunologically “cold” or “excluded” tumors is the rational combination of ICIs with Antibody-Drug Conjugates (ADCs). ADCs targeting surface antigens overexpressed in HNSCC-, such as EGFR, Human Epidermal Growth Factor Receptor 3 (HER3), or the immune checkpoint B7-H3-, utilize highly selective monoclonal antibodies to deliver highly potent cytotoxic payloads directly inside the malignant cell [41,42]. Beyond direct tumor cell lysis, modern ADCs utilize membrane-permeable payloads that diffuse into neighboring tumor cells, bypassing heterogeneous antigen expression (the bystander effect) [43]. While targeted destruction can potentially induce immunogenic cell death (ICD) and stimulate the release of Damage-Associated Molecular Patterns (DAMPs) alongside structural tumor neoantigens [43], this phenomenon is payload-dependent and not universally induced across all ADC platforms.

### 8.3. Therapeutic Vaccines and Cellular Therapies

In virus-associated HNSCC and NPC, targeting specific, non-self viral antigens remains an ideal strategy to achieve high therapeutic precision while minimizing off-target toxicities. Active clinical trials are investigating therapeutic DNA, RNA, and peptide-based vaccines targeting the E6 and E7 oncoproteins of high-risk HPV16/18, or the latent membrane proteins (LMP1/LMP2) of EBV [42]. When combined with systemic anti-PD-1 ICIs, these vaccines induce the clonal expansion of virus-specific, naive T cells within peripheral lymphoid organs, ensuring a continuous supply of highly targeted, non-exhausted cytotoxic T cells to the tumor site, effectively replicating the biological architecture found naturally in exceptional responders [20,44]. Concurrently, adoptive cell transfer strategies, including Chimeric Antigen Receptor (CAR)-T cells, TCR-engineered T cells, and the expansion of Tumor-Infiltrating Lymphocytes (TILs), represent a highly personalized modality designed to overwhelm the protective immunosuppressive shield of advanced head and neck tumors [45]. However, the broader clinical translation of adoptive cellular therapies in HNSCC remains constrained by significant logistical and biological hurdles: these approaches are currently limited to early phases, where efficacy is challenged by marked intratumoral antigen heterogeneity, poor T-cell trafficking into solid tumor stroma, and substantial safety risks, including cytokine release syndrome (CRS), neurotoxicity, and off-target on-tissue destruction [45,46].

### 8.4. Evidence-Graded Overview of Emerging Platforms

To provide a structured synthesis of the emerging therapeutic paradigm, the clinical development stages, targets, observed efficacy signals, safety considerations, and major ongoing limitations of these novel platforms are summarized below in Table 3.

## 9. Conclusions

Immunotherapy targeting the PD-1/PD-L1 axis has established a vital foundation in the management of recurrent or metastatic head and neck oncology, offering a subset of patients long-term survival benefits and an improved tolerability profile compared to traditional cytotoxic chemotherapy. However, unselected clinical deployment yields modest overall response rates, underscoring an urgent clinical imperative to transition from unselected clinical utilization to biomarker-driven personalization and dynamic patient stratification.

As clearly illustrated by exceptional responder profiles—such as the complete resolution of ultra-high-mortality stomal recurrences—long-term success relies on a critical configuration of high baseline immune inflammation, functional antigen presentation pathways, high antigenic novelty, and microenvironmental patency. Future clinical efforts must leverage these insights when evaluating emerging combinatorial regimens, combining next-generation checkpoint blockades, tumor-targeted ADCs that induce immunogenic cell death, and virus-or-antigen-specific immunotherapies within rigorously designed, biomarker-enriched clinical trials. Crucially, the clinical translation of these novel platforms will require balancing potential synergistic efficacy against additive treatment-emergent toxicities, manufacturing constraints, and marked intratumoral heterogeneity to safely expand durable responses across a broader HNSCC population.

## Figures and Tables

**Table 1:** Efficacy outcomes from the KEYNOTE-048 study in R/M HNSCC. The “+” notation indicates a censored observation, representing an ongoing response at the time of the data cutoff.

Population/Population Subset	Treatment Arm	Overall Survival (OS) [Median, Mo (95% CI)]	Progression-Free Survival (PFS) [Median, Mo (95% CI)]	Objective Response Rate (ORR) [% (95% CI)]	Duration of Response (DOR) [Median, Mo (Range)]
**CPS** ≥ **20**	Pembro Alone	**14.9** (11.6–17.8)	**3.4** (3.2–4.9)	**23.3%** (16.9–30.9)	**22.6** (1.5+ to 43.0+)
	Pembro + Chemo	**14.7** (11.3–19.5)	**5.8** (4.7–7.6)	**42.9%** (35.2–50.9)	**7.1** (1.6+ to 39.0+)
	EXTREME Control	**10.7** (9.3–12.1)	**5.2** (4.9–6.0)	**36.1%** (28.5–44.3)	**4.2** (1.2+ to 38.6+)
**CPS** ≥ **1**	Pembro Alone	**12.3** (10.8–14.9)	**3.2** (2.2–3.4)	**19.1%** (14.9–23.9)	**23.4** (1.5+ to 43.0+)
	Pembro + Chemo	**13.6** (11.1–15.7)	**5.1** (4.7–6.2)	**36.4%** (30.7–42.3)	**6.7** (1.6+ to 39.0+)
	EXTREME Control	**10.3** (9.0–11.5)	**5.0** (4.8–6.0)	**35.7%** (30.1–41.6)	**4.5** (1.2+ to 38.6+)
**Total** **Population**	Pembro Alone	**11.5** (10.3–13.4)	**2.3** (2.2–3.3)	**16.9%** (13.3–21.0)	**22.6** (1.5+ to 43.0+)
	Pembro + Chemo	**13.0** (10.9–14.7)	**4.9** (4.7–6.0)	**35.6%** (30.3–41.1)	**6.7** (1.6+ to 39.0+)
	EXTREME Control	**10.7** (9.3–11.7)	**5.1** (4.9–6.0)	**36.3%** (31.0–41.9)	**4.5** (1.2+ to 38.6+)

**Table 2:** Proposed working hypothesis of biological, molecular, and microenvironmental drivers associated with exceptional response to immune checkpoint inhibitors in HNSCC. Abbreviations: Trm, tissue-resident memory T cells; MDSCs, myeloid-derived suppressor cells; M2 TAM, M2-polarized tumor-associated macrophages; *B2M*, beta-2-microglobulin; *TAP1/TAP2*, transporter associated with antigen processing ½.

Core Biological Axis	Specific Molecular Feature	Mechanistic Consequence in TME	Clinical Relevance	Evidence Base & Nature of Data
**Immune** **Infiltration** **Status**	High core density of CD8+ and CD103+ Trm cells.	High baseline secretion of *IFN-gamma* and granzyme B; immune “hot” profile.	Immediate tumor cytoreduction upon PD-1 axis disinhibition.	Directly observed in HPV+ HNSCC exceptional responder cohorts exhibiting “hot” TME and tissue-resident memory T cells.
**Antigen** **Presentation** **Integrity**	Wild-type (unmutated) *B2M*, *TAP1*, *TAP2*, and *JAK1/JAK2* loci.	Preserved intracellular transport and stable surface presentation of peptides.	Reduced risk of “immune blindness” or acquired evasion.	Working hypothesis extrapolated from established escape/resistance mechanisms in pretreated HNSCC.
**Antigenic** **Novelty Profile**	Clonal viral oncoproteins (HPV E6/E7; EBV LMP1) OR high tobacco frameshift indels.	Generation of continuous, immunogenic non-self novel antigens.	Robust activation of T-cell receptors without central tolerance check.	Directly measured in viral-driven (HPV/EBV) elite responders and high-indel tobacco-induced cohorts.
**Microenvironmental** **Architecture**	Low concentrations of MDSCs, M2 TAMs, and dense cross-linked collagen stroma.	Reduced physical and immunosuppressive barriers within the matrix.	Facilitated migration of cytotoxic lymphocytes and delivery of therapeutic mAbs.	Extrapolated from general HNSCC resistance and immune-exclusion studies showing physical stromal barriers.

**Table 3:** Overview of emerging immunotherapeutic modalities, specific representative agents, target pathways, clinical phases, efficacy signals, safety considerations, and limitations in HNSCC. Abbreviations: ADCs, antibody-drug conjugates; NGCIs, next-generation checkpoint inhibitors; ICD, immunogenic cell death; CRS, cytokine release syndrome; TCR-T, T-cell receptor-engineered T cells; ICIs, immune checkpoint inhibitors; TME, tumor microenvironment.

Platform	Specific Representative Agents and Target Pathway/Antigen	Trial Phase and Primary Clinical Trial (NCT Number)	Target HNSCC Population	Efficacy and Safety Findings	Key Limitations and Current Status
**Next-Generation Checkpoint Inhibitors (NGCIs)**	• Relatlimab (LAG-3) • Sabatolimab (TIM-3)	Phase I/II • NCT01968109 (Relatlimab) • NCT02608268 (Sabatolimab)	Advanced or platinum-refractory recurrent/metastatic HNSCC	Modest single-agent activity; aims to re-energize exhausted T cells in mucosal TME	Mixed/variable efficacy signals; require biomarker selection rather than unselected cohort enrollment
**Antibody-Drug Conjugates (ADCs)**	• Tisotumab Vedotin (Tissue Factor) • Sacituzumab Govitecan (TROP-2) • BL-B01D1 (EGFR/HER3 Bispecific)	Phase I/II • NCT03485209 (Tisotumab) • NCT03964727 (Sacituzumab) • NCT05160125 (BL-B01D1)	Pretreated recurrent/metastatic HNSCC progressing after platinum-based chemotherapy and anti-PD-1/PD-L1 immunotherapy	Targeted tumor lysis and bystander effect; potential induction of immunogenic cell death (ICD)	Potential ICD is payload-dependent; risk of severe toxicities including neutropenia, neuropathy, interstitial lung disease, and ocular toxicities
**Therapeutic Cancer Vaccines**	• ISA101/ISA101b (HPV16 E6/E7) • TG4001 (HPV16 E6/E7) • mRNA-4157 (V940) (Patient-specific Neoantigens)	Phase I/II • NCT02426892 (ISA101) • NCT03260023 (TG4001) • NCT03897881 (mRNA-4157)	HPV16-positive recurrent/metastatic HNSCC (for viral vaccines); adjuvant high-risk resected HNSCC (for mRNA-4157)	Clonal expansion of naive virus-specific T cells; high precision with low off-target toxicity	Requires combination with ICIs for optimal activity; efficacy constrained in non-viral tumors
**Adoptive Cellular Therapies**	• E6/E7 TCR-T (HPV-16 E6/E7)	Phase I/II • NCT02858310 (E6/E7 TCR-T)	Advanced or refractory HPV-positive HNSCC progressing after multiple lines of systemic therapy and standard immunotherapy	Personalized T-cell reactivity against viral antigens	High risk of cytokine release syndrome (CRS), neurotoxicity, and off-target toxicity; limited by intratumoral heterogeneity and stroma trafficking

## Data Availability

The original contributions presented in this study are included in the article. Further inquiries can be directed to the corresponding author.
